# Cerebral Microstructural Alterations in Patients With Early Parkinson’s Disease Detected With Quantitative Magnetic Resonance Measurements

**DOI:** 10.3389/fnagi.2021.763331

**Published:** 2021-11-01

**Authors:** Martin Klietz, M. Handan Elaman, Nima Mahmoudi, Patrick Nösel, Mareike Ahlswede, Florian Wegner, Günter U. Höglinger, Heinrich Lanfermann, Xiao-Qi Ding

**Affiliations:** ^1^Department of Neurology, Hannover Medical School, Hanover, Germany; ^2^Institute of Diagnostic and Interventional Neuroradiology, Hannover Medical School, Hanover, Germany

**Keywords:** Parkinson’s disease, relative proton density, T1 relaxation time, T2 relaxation time, T2′ relaxation time, quantitative MRI analysis, biomarker, early diagnosis

## Abstract

**Objective:** Parkinson’s disease (PD) is the second most common neurodegenerative disease in the elderly. In early stages of PD, patients typically display normal brain magnet resonance imaging (MRI) in routine screening. Advanced imaging approaches are necessary to discriminate early PD patients from healthy controls. In this study, microstructural changes in relevant brain regions of early PD patients were investigated by using quantitative MRI methods.

**Methods:** Cerebral MRI at 3T was performed on 20 PD patients in early stages and 20 age and sex matched healthy controls. Brain relative proton density, T1, T2, and T2′ relaxation times were measured in 14 regions of interest (ROIs) in each hemisphere and compared between patients and controls to estimate PD related alterations.

**Results:** In comparison to matched healthy controls, the PD patients revealed decreased relative proton density in contralateral prefrontal subcortical area, upper and lower pons, in ipsilateral globus pallidus, and bilaterally in splenium corporis callosi, caudate nucleus, putamen, thalamus, and mesencephalon. The T1 relaxation time was increased in contralateral prefrontal subcortical area and centrum semiovale, putamen, nucleus caudatus and mesencephalon, whereas T2 relaxation time was elevated in upper pons bilaterally and in centrum semiovale ipsilaterally. T2′ relaxation time did not show significant changes.

**Conclusion:** Early Parkinson’s disease is associated with a distinct profile of brain microstructural changes which may relate to clinical symptoms. The quantitative MR method used in this study may be useful in early diagnosis of Parkinson’s disease. Limitations of this study include a small sample size and manual selection of the ROIs. Atlas-based or statistical mapping methods would be an alternative for an objective evaluation. More studies are necessary to validate the measurement methods for clinical use in diagnostics of early Parkinson’s disease.

## Introduction

The prevalence of Parkinson’s disease (PD) in Germany is about 0.5% of the population, according to recently published data, which seems to be representative of industrial countries worldwide (Heinzel et al., 2018). Patients with PD experience a pronounced decrease in quality of life in the course of the disease (Klietz et al., 2018, 2020).

The main pathological processes in PD are known to include the deposition of Lewy bodies across the brain in a specific stage-related manner, and the loss of dopaminergic neurons innervating the basal forebrain *via* long unmyelinated axons (Halliday and McCann, 2010; Kalia and Kalia, 2015; Rai and Singh, 2020; Rai et al., 2020, 2021). This results in dopamine depletion in the basal forebrain and further maladaptive changes in neurotransmission, for example in striatal interneurons (Keber et al., 2015; Klietz et al., 2016).

Braak et al. (2004) described brain Lewy body pathology as an ascending process beginning in the dorsal nucleus of the vagal nerve in stage I, reaching substantia nigra dopaminergic neurons in stage III and, finally, covering the majority of the cerebral cortex in stages V and VI (Braak et al., 2004; Rai et al., 2016, 2017; Braak and Tredici, 2017). Despite a few weaknesses, the Braak model is still the most widely accepted theory of progression of Lewy pathology in PD (Jellinger, 2019). Alpha-synuclein is a relevant component of Lewy bodies and there is growing evidence showing a spread of misfolded oligomeric alpha-synuclein fibrils over the brain contributing to PD progression *via* neuronal toxicity (Olanow and Prusiner, 2009). Alpha-synuclein unfolds its toxic properties in various cell components. First, alpha-synuclein impairs the mitochondrial function (Sheng and Cai, 2012). Additionally, it disturbs intracellular protein homeostasis, leading to chronic endoplasmic reticulum stress (Burré et al., 2018). Further, it can form pore-like structures that perforate membranes including the plasma membrane (Stöckl et al., 2013; Angelova and Abramov, 2017). Several studies also describe mild inflammatory reactions as a component of PD pathology (Zhang et al., 2005; Alvarez-Erviti et al., 2011; Depboylu et al., 2011a, b).

In the differential diagnosis of PD, magnetic resonance imaging (MRI) is an important tool (Meijer and Goraj, 2014). However, PD patients typically present a normal routine MRI scan and only patients with atypical Parkinsonism may show specific imaging features (Yekhlef et al., 2003). Since standard MRI measures do not discriminate healthy controls from PD patients, advanced imaging methods are needed for detection of PD related brain changes with higher sensitivity (Huppertz et al., 2016; Fu et al., 2020). Therefore, patients at different PD stages were studied with combined multiple parametric MRI and MR spectroscopic imaging methods (Klietz et al., 2019a; Fu et al., 2020). In this study, quantitative MRI measurements were used to study PD patients at an early stage.

Quantitative MRI (qMRI) measurements could be used to determine brain tissue parameters, such as relative proton density that is related to tissue free water content, relaxation time T1 characterizing the longitudinal relaxation process, T2, T2′, and T2^∗^ that describe the irreversible (T2), reversible (T2′) and apparent transverse relaxation processes (T2^∗^) (Eylers et al., 2016; Gracien et al., 2019). Pathological conditions may cause altered values of these parameters: Proton density changes, for example due to edema (Jurcoane et al., 2013; Carey et al., 2018; Tofts, 2018; Filo et al., 2019). T1, T2, T2^∗^, and T2′ relaxation times may provide information about various tissue properties like water and iron content, degree of myelination, axonal damage, and gliosis (Fatouros et al., 1991; Gelman et al., 1999, 2001; Brex et al., 2000; Laule et al., 2004; Lutti et al., 2014; Waehnert et al., 2016), as well as the local concentration of deoxyhemoglobin or metabolic activity of the brain tissue (Wagner et al., 2012; Eylers et al., 2016). Therefore, qMRI measurement may be a potential tool for detection of early brain changes in PD patients. Recent studies in Alzheimer’s disease showed similarly that detection of early cerebral microstructural changes using MRI relaxation methods can be useful in the early diagnosis of the disease, potentially providing a non-invasive biomarker for Alzheimer’s disease which can also predict cognitive decline in the course of disease development (Reas et al., 2018; Wearn et al., 2020).

Until now, few structured 3T qMRI analyses of a carefully characterized PD cohort compared to healthy controls are available (Langkammer et al., 2016; Nürnberger et al., 2017; Chen et al., 2019). The aim of the present study is to estimate the feasibility of using qMRI measurements to detect brain changes in patients with early PD.

## Patients and Methods

### Patients and Clinical Examinations

This study was conducted with ethical approval of the local Ethics Committee of Hannover Medical School (No. 6167-2016). All patients and participants gave their written informed consent. Patients were recruited from the neurological wards, the outpatient clinic and from local patient support groups. The diagnosis of PD was confirmed according to the Movement Disorder Society (MDS) diagnostic criteria for PD by a movement disorder specialist (Postuma et al., 2015). For none of the PD patients an alternative diagnosis was more likely than PD.

Only early-stage PD patients, who were scaled clinically with Hoehn and Yahr stages (H&Y) of 1 or 2 in the best medical “on” state and aged 75 or below, were included in the study. Definition of early-stage PD by the H&Y stages is in accordance with earlier studies of our group (Klietz et al., 2019a; Fu et al., 2020), additionally, none of our patients complained of a significant amount of motor complications qualifying for advanced PD (Titova et al., 2017). Patients with atypical Parkinsonism and other known brain pathologies, e.g., stroke, small vessel disease, or tumor, were not included in this study. Patients with clinical conditions interfering with MRI diagnostics, like severe head tremor, dystonia or dyskinesia, had to be excluded as well. PD patients with severe comorbidities were also excluded from our study (Klietz et al., 2019b; Greten et al., 2020).

Twenty PD patients (48–72 years old, mean age 60.2 ± 7.2 years, 12 females) were included. Disease specific information like disease duration, dominantly affected body side, predominant symptoms, general and PD medication and comorbidities were documented. 16 of 20 PD patients underwent a dopamine transporter (DAT) scan in the diagnostic work up in routine care, in all cases the DAT scan revealed lateralized nigro-striatal dopaminergic neurodegeneration. PD specific symptoms were assessed by the Movement Disorder Society Unified Parkinson’s Disease Rating Scale (MDS-UPDRS) (Goetz et al., 2008). Patients were rated in best medication “on” state. PD specific medication was noted and levodopa equivalence dosage calculated (LED) (Tomlinson et al., 2010). Cognitive deficits were quantified by the established test for dementia and mild cognitive impairment “DemTect” (Kalbe et al., 2004). 20 healthy participants matched in age and sex on a one-to-one basis were also studied as the control cohort. All patients and healthy controls were right-handed according to self-report.

Part of this early PD patient cohort was studied previously with other MRI methods (Klietz et al., 2019a; Fu et al., 2020). Patient characteristics showed some differences as one patient was replaced due to incomplete MRI scans.

### MR Examinations and Data Processing

All subjects underwent MR examinations at 3T (Verio, Siemens, Erlangen, Germany), with the routine MRI protocol including the following sequences for subsequent qMRI measurements: a T2 weighted turbo spin echo (T2-TSE) sequence with three echoes (triple TE) (TR/TE = 6640/8.7/70/131 ms; 150° flip angle; 256 × 208 matrix; 1 mm × 1 mm × 3 mm voxel size), a T2 weighted gradient echo (T2-GRE) sequence with triple TE (TR/TE = 1410/6.42/18.42/30.42 ms; 20° flip angle; 256 × 208 matrix; 1 mm × 1 mm × 3 mm voxel size), a T1 weighted three-dimensional GRE (T1-GRE) sequence with two flip angles (T1wGRE, TR/TE = 15/1.64 ms, flip angles 5° and 25°; 256 × 208 matrix; 1 mm × 1 mm × 3 mm voxel size), and a fluid attenuation inversion recovery (FLAIR) sequence. The scans were obtained with the same angulation enabling identifications of the same brain structures. An aqueous phantom was also scanned with the Triple TE sequence to derive proton density of the phantom (PD_*H2O*_), which was used for normalization of the proton density measured from subjects.

The inspection of FLAIR, TSE and GRE images to identify possible morphological abnormalities was completed by two independent neuroradiologists. Participants with morphological MRI abnormalities were excluded from this study. Parameter maps of the relaxation times T1, T2, and T2^∗^ were obtained on-the-fly by the MR console with an extended image reconstruction, provided by the manufacturer, with monoexponentially fitting to the signal-intensity decay curves based on the data acquired with T1-GRE, T2-TSE and T2-GRE sequences, respectively. The maps of T2′ were derived according to the equation 1/T2′ = 1/T2 + 1/T2^∗^. The derived brain maps of proton density, T1, T2, and T2′ were used for region of interest analysis.

### Region of Interest Analysis

**FIGURE 1:**
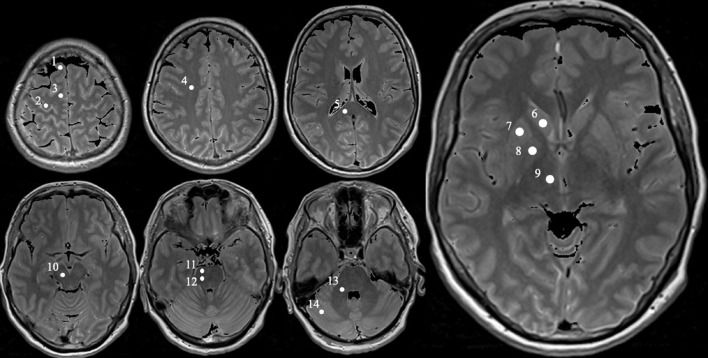
Exemplary MRI with definition of the specific measured regions obtained from a PD patient (female, 62). Positions of each selected ROI in the right brain hemisphere marked as white circle on proton density weighted MR images obtained from a 62-year-old female Parkinson’s disease patient. The circles are numbered 1 to 14, representing the following ROIs: prefrontal subcortical area (1), subcortical primary motor area (2), supplementary motor area (3), centrum semiovale (4), splenium corporis callosi (5), nucleus caudatus (6), putamen (7), globus pallidus (8), thalamus (9), mesencephalon (containing substantia nigra and ventral tegmental area, 10), upper and lower pons (11 and 12, respectively), cerebellar white matter (13), and cerebellar posterior lobe (14).

In each subject, the values of proton density, T1, T2, and T2′ were determined by using mean values over a region of interest (ROI). In total, 28 ROIs (14 in each brain hemisphere) were selected in consideration of their neuroanatomical functions and the involvement in Parkinson’s disease, and were located bilaterally in the prefrontal subcortical area, subcortical primary motor area, supplementary motor area, centrum semiovale, splenium corporis callosi, nucleus caudatus, putamen, globus pallidus, thalamus, mesencephalon (containing substantia nigra and ventral tegmental area), upper and lower pons, cerebellar white matter, and cerebellar posterior lobe (Figure 1). The relevant substantia nigra area is very small so that a ROI covering only this part would lead to erroneous measurements due to unavoidable partial volume effects. Hence, a ROI with a larger area in the ventral mesencephalon covering the substantia nigra fully was selected and named “mesencephalon,” as this region covers also the surrounding midbrain parts in addition to the substantia nigra.

**FIGURE 2:**
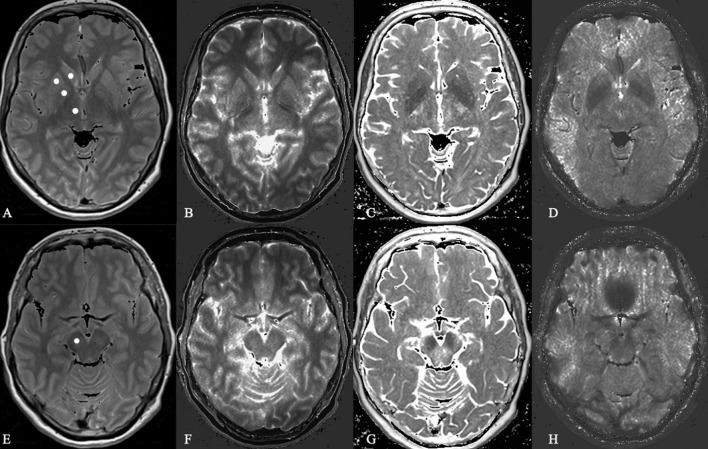
Microstructural analysis of proton density, T1 relaxation time, T2 and T2 prime in a Parkinson’s disease patient. In this Figure shows the basal ganglia **(A–D)** and mesencephalon **(E–H)** in proton density-weighted **(A,E)**, T1-weighted **(B,F)**, T2-weighted **(C,G)**, and T2 prime-weighted **(D,H)** MR sequences obtained from a 62-year-old female PD patient.

All ROIs were carefully drawn manually as a circle of about 20 mm^2^ according to anatomical landmarks. Subsequently, the values of relative proton density (= measured value/PD_*H2O*_ presented in%), T1, T2, and T2′ were obtained from each of the ROIs (Figure 2). All derived data were controlled by the following quality criteria: Values of the ROIs measured from brain tissue parameter maps with a coefficient of variation (= standard deviation/mean value, COV) higher than 25% were excluded from further statistical analysis.

### Statistical Analysis

The measured data were statistically compared between patients and healthy controls. The data measured from left and right brain hemispheres of the PD patients were rearranged into data of contralateral or ipsilateral hemispheres in regard to the symptom onset body side for the comparison. In order to minimize possible bias related to natural hemisphere laterality in the human brain, we used hemisphere-matched data of healthy controls as reference data. For example, for a PD patient with right dominant clinical symptoms, the right cerebral hemisphere is ipsilateral to the clinically pronounced side. Accordingly, in the healthy volunteer the right hemisphere was viewed ipsilateral and the left as contralateral, for conformity. After the normality of the data distribution was checked with Shapiro–Wilk test and quantile-quantile plots, a paired *t*-test was used to compare the measured values between patients and the matched healthy controls. The comparison was made both for the data of the contralateral and the data of the ipsilateral hemisphere. False-discovery rate (FDR) with a desired false-discovery rate of 0.05 was used for multiple comparison correction (Benjamini and Hochberg, 1995), with a corresponding FDR corrected significance threshold of pi = 0.05/i, i varied from n, n−1, … to 1, where, *n* = 14, the number of multiple testing that was equal to the number of the ROIs in each hemisphere. Results with a p less than 0.05 were controlled with FDR, where those with a p less than the corresponding pi were considered significant cases, and those with a p larger than the corresponding pi were considered not significant. Spearman’s correlation test with a significance level of 0.05 was used to estimate possible correlations between clinical MDS-UPDRS scores and significantly altered brain parameter values in PD patients.

Statistical analyses were performed with SPSS version 23 (SPSS IBM, New York, United States).

## Results

### Parkinson’s Disease Patient Characteristics

20 early stage clinically diagnosed PD subjects were included in this study. None of the patients were suspected to suffer from atypical Parkinsonism or were cognitively impaired. All patients reported an obviously positive response to dopaminergic treatment and were examined in the best medication “on” state. All PD patient characteristics are displayed in Table 1. All PD subjects revealed good cognitive function measured by the DemTect with a mean score of 16.0 (SD 1.9, min 13 and max 18). The patients displayed a mean value of 7.4 (SD 4.4, min 2, max 17) in MDS-UPDRS part II as motor aspects of daily living. Motor deficits in the best medication “on” state of our PD subjects in the MDS-UPDRS part III scored a mean of 16.5 points (SD 7.9, min 5 and max 31).

**TABLE 1:** Patient characteristics.

	Mean	SD	Min	Max
12 females, 8 males				
Clinically dominant side: 12 right, 8 left				
Type: 8 ET, 8 TD, 4 AR				
H&Y stage	1.8	0.5	1	2
Disease duration (in years)	6.0	3.7	1	13
MDS-UPDRS part I	7.7	4.5	2	20
MDS-UPDRS part II	7.4	4.4	2	17
MDS-UPDRS part III	16.5	7.9	5	31
MDS-UPDRS part IV	0.4	1.1	0	4
DemTect	16.0	1.9	13	18
LED	770 mg	521 mg	0 mg	1,600 mg

*H&Y Hoehn and Yahr stage; MDS-UPDRS Movement Disorders Society Unified Parkinson’s Disease Rating Scale; DemTect test for cognitive assessment; ET equivalence type; TD tremor dominant; AR akinetic-rigid; LED Levodopa-equivalence dosage.*

### Quantitative MR Measurements

Tables 2–5 show the results of paired *t*-tests between PD patients and matched controls for relative proton densities, T1, T2, and T2′, measured from contralateral and ipsilateral hemispheres of the patients in relation to the clinically affected/more affected body hemisphere as well as from the corresponding matched hemisphere of the healthy controls.

**TABLE 2:** Paired *t*-tests between patients and controls for relative proton densities measured in brain hemispheres contralateral or ipsilateral to the more affected body side as indicated.

Region of Interest		Ipsilateral					Contralateral				
	*N* ^1^	Patient		Control		*p*	Patient		Control		*p*
		Mean	SD	Mean	SD		Mean	SD	Mean	SD	
Prefrontal Subcortical Area	19	49.78	3.72	47.14	4.99	0.07	50.47	3.14	46.77	3.74	0.0024**
Subcortical Primary Motor Area	20	44.02	2.85	46.88	4.35	0.011*	44.63	3.45	44.98	3.53	0.78
Supplementary Motor Area	20	50.23	4.09	50.10	6.22	0.92	53.13	5.05	50.76	5.58	0.11
Centrum Semiovale	20	42.78	2.39	44.68	2.91	0.011*	42.55	3.20	44.37	2.76	0.06
Splenium Corporis Callosi	20	35.28	3.42	38.66	3.24	0.0002**	35.16	2.83	39.07	3.49	0.00003**
Nucleus Caudatus	20	51.46	3.48	54.70	3.28	0.0018**	50.88	3.28	55.02	3.85	0.0013**
Putamen	20	50.40	4.30	54.07	3.63	0.002**	51.06	2.55	54.44	3.61	0.0016**
Globus Pallidus	20	41.63	3.70	45.22	3.27	0.0009**	41.26	3.34	44.32	3.34	0.0154*
Thalamus	20	43.46	3.21	48.79	4.27	0.00002**	43.32	2.92	49.14	3.53	0.00002**
Mesencephalon	20	36.35	3.09	40.10	4.39	0.0023**	36.22	2.86	39.96	3.80	0.0041**
Upper Pons	20	30.81	3.24	33.01	3.07	0.0182*	30.99	2.16	33.12	2.66	0.0062**
Lower Pons	20	33.03	4.30	35.46	3.57	0.0174*	32.84	2.71	35.69	3.17	0.0052**
Cerebellar White Matter	20	34.79	3.54	35.32	3.77	0.57	35.08	2.05	35.51	3.58	0.57
Cerebellar Posterior Lobe	20	49.35	5.10	51.05	3.57	0.18	49.76	4.63	48.95	5.60	0.58

*^1^Number of patient-control pairs.*

**Significant after correction for multiple comparisons by using false-discovery rate (FDR).

*p < 0.05 but not significant after FDR correction.

In comparison to matched healthy controls, our patients revealed significant decreases (*p* < 0.05 and pi) of brain relative proton density in 8 of 14 contralateral ROIs (contralateral prefrontal subcortical area, splenium corporis callosum, caudate nucleus, putamen, thalamus, mesencephalon, upper and lower pons), and 6 of 14 ipsilateral ROIs (ipsilateral splenium corporis callosum, caudate nucleus, putamen, globus pallidus, thalamus, and mesencephalon), while showing a trend to decrease (pi < *p* < 0.05, not significant after correction for multiple testing) in five ROIs, including contralateral globus pallidus, and ipsilateral subcortical primary motor area, centrum semiovale, upper and lower pons (Table 2).

Compared to healthy controls, our PD patients also revealed a significant increase of T1 relaxation time (*p* < 0.05 and pi) in three contralateral ROIs and in one ipsilateral ROI (contralateral prefrontal subcortical area, centrum semiovale, splenium corpus callosum, and ipsilateral centrum semiovale). Additionally, the data showed a trend to increase (pi < *p* < 0.05, not significant after correction for multiple testing) in five contralateral and four ipsilateral ROIs (contralateral subcortical primary motor area, supplementary motor area, caudate nucleus, putamen, and mesencephalon, and ipsilateral prefrontal subcortical area, splenium corpus callosum, putamen, and upper pons, Table 3).

**TABLE 3:** Paired *t*-tests between patients and controls for T1 relaxation times measured in brain hemisphere contralateral or ipsilateral to the more affected body side as indicated.

Region of Interest		Ipsilateral					Contralateral				
	*N* ^1^	Patient		Control		*p*	Patient		Control		*p*
		Mean	SD	Mean	SD		Mean	SD	Mean	SD	
Prefrontal Subcortical Area	19	1, 026.79	191.32	849.45	159.89	0.0085*	1, 059.11	184.49	869.33	123.54	0.0011**
Subcortical Primary Motor Area	20	995.67	129.37	938.36	71.28	0.078	993.04	97.61	936.28	128.63	0.0488*
Supplementary Motor Area	19	1, 187.13	235.93	1, 064.81	249.25	0.19	1, 232.19	196.85	1, 055.84	289.86	0.0097*
Centrum Semiovale	20	1, 126.00	120.97	995.42	90.25	0.0011**	1, 118.82	119.01	996.74	120.52	0.0008**
Splenium Corporis Callosi	20	1, 420.66	123.40	1, 293.15	148.74	0.0052*	1, 422.51	142.67	1, 279.92	153.91	0.0023**
Nucleus Caudatus	20	2, 002.53	183.38	1, 938.72	188.31	0.27	2, 111.10	169.36	1, 978.04	148.02	0.0177*
Putamen	20	1, 933.18	173.99	1, 795.14	139.92	0.0102*	1, 974.17	164.92	1, 825.39	200.67	0.0203*
Globus Pallidus	20	1, 612.88	219.98	1, 548.07	174.52	0.33	1, 634.94	172.93	1, 563.41	211.27	0.24
Thalamus	20	2, 443.62	331.26	2, 375.49	256.63	0.47	2, 384.40	307.89	2, 309.73	338.12	0.4
Mesencephalon	19	1, 863.19	246.24	1, 772.37	162.36	0.17	1, 861.59	151.66	1, 747.23	180.17	0.0391*
Upper Pons	20	1, 863.93	186.70	1, 737.71	156.43	0.0172*	1, 845.78	192.32	1, 839.76	181.69	0.9
Lower Pons	20	1, 981.60	265.19	1, 876.63	191.22	0.16	1, 893.47	205.50	1, 843.12	235.43	0.4
Cerebellar White Matter	20	1, 693.94	178.22	1, 606.62	182.62	0.15	1, 535.96	183.33	1, 572.04	194.26	0.49
Cerebellar Posterior Lobe	20	1, 678.96	312.26	1, 669.21	186.02	0.9	1, 471.78	270.08	1, 473.91	262.18	0.98

*^1^Number of patient-control pairs.*

**Significant after correction for multiple comparisons by using false-discovery rate (FDR).

*p < 0.05 but not significant after FDR correction.

Furthermore, in comparison to healthy controls, the PD patients revealed a significant increase of T2 relaxation time (*p* < 0.05 and pi) in one contralateral and 2 ipsilateral ROIs (contralateral upper pons, and ipsilateral splenium corpus callosum and upper pons), and showed a trend to increase (pi < *p* < 0.05, not significant after correction for multiple testing) in ipsilateral cerebellar white matter (Table 4).

**TABLE 4:** Paired *t*-tests between patients and controls for brain T2 relaxation times measured in brain hemisphere contralateral or ipsilateral to the more affected body side as indicated.

Region of Interest		Ipsilateral					Contralateral				
	*N* ^1^	Patient		Control		*p*	Patient		Control		*p*
		Mean	SD	Mean	SD		Mean	SD	Mean	SD	
Prefrontal Subcortical Area	14	137.32	16.57	146.20	18.29	0.22	132.25	22.50	142.55	16.30	0.14
Subcortical Primary Motor Area	19	123.38	9.54	119.49	13.80	0.35	116.70	12.68	121.50	10.83	0.27
Supplementary Motor Area	19	111.63	14.26	119.77	10.74	0.07	116.23	19.30	123.29	12.39	0.24
Centrum Semiovale	20	124.06	9.70	127.91	7.52	0.16	123.49	11.45	127.12	9.21	0.3
Splenium Corporis Callosi	19	124.53	17.13	107.49	9.99	0.0005**	118.14	25.42	107.41	9.92	0.09
Nucleus Caudatus	19	98.66	8.75	100.13	5.28	0.58	101.71	8.00	99.86	5.74	0.47
Putamen	20	91.29	7.07	87.50	5.90	0.07	89.39	6.39	88.41	5.89	0.58
Globus Pallidus	18	75.20	5.19	72.99	5.18	0.2	73.95	9.37	74.95	7.53	0.74
Thalamus	20	108.63	10.94	104.51	5.22	0.12	107.27	7.64	104.52	8.35	0.27
Mesencephalon	18	94.70	8.97	94.90	14.16	0.9	93.05	9.06	92.97	8.47	0.98
Upper Pons	20	129.84	13.85	115.53	7.97	0.0011**	132.30	20.47	114.64	8.17	0.0013**
Lower Pons	17	126.65	21.00	124.91	17.44	0.8	123.86	15.58	120.21	14.33	0.44
Cerebellar White Matter	20	142.69	16.88	132.07	6.62	0.0145*	140.88	18.43	133.61	9.58	0.12
Cerebellar Posterior Lobe	17	137.90	15.20	136.88	11.28	0.79	143.65	24.95	131.93	13.88	0.08

*^1^Number of patient-control pairs.*

**Significant after correction for multiple comparisons by using false-discovery rate (FDR).

*p < 0.05 but not significant after FDR correction.

No significant difference between patients and healthy controls was found in T2′ relaxation time, while a trend to altered T2′ (*p* < 0.05 and pi) was found in four contralateral ROIs of the PD patients, including a trend to increase in contralateral splenium corpus callosum and globus pallidus, and a trend to decrease in contralateral supplementary motor area and mesencephalon (Table 5).

**TABLE 5:** Paired *t*-tests between patients and controls for T2′ measured in brain hemisphere contralateral or ipsilateral to the more affected body side as indicated.

Region of Interest		Ipsilateral					Contralateral				
	*N* ^1^	Patient		Control		*p*	Patient		Control		*p*
		Mean	SD	Mean	SD		Mean	SD	Mean	SD	
Prefrontal Subcortical Area	5	81.16	17.06	83.45	12.09	0.85	84.05	12.91	83.60	12.66	0.94
Subcortical Primary Motor Area	17	56.00	7.27	57.10	10.60	0.70	60.73	10.73	59.03	8.00	0.63
Supplementary Motor Area	13	66.68	15.24	64.86	9.75	0.67	67.57	8.51	74.06	9.21	0.0214*
Centrum Semiovale	17	69.58	8.41	69.53	7.31	0.99	70.72	10.79	69.64	11.34	0.77
Splenium Corporis Callosi	18	53.85	12.63	51.68	7.62	0.40	55.92	11.71	48.51	7.26	0.024*
Nucleus Caudatus	16	57.79	12.06	56.19	8.28	0.65	58.62	18.24	61.98	15.36	0.63
Putamen	17	47.21	9.53	49.73	14.36	0.53	49.56	11.51	44.67	8.44	0.21
Globus Pallidus	10	36.15	5.15	33.84	8.85	0.56	34.68	6.20	27.32	5.81	0.0054*
Thalamus	13	69.80	11.49	71.41	8.05	0.69	73.98	9.72	74.88	14.18	0.86
Mesencephalon	5	30.22	7.01	28.39	10.06	0.80	28.59	8.10	37.24	8.20	0.035*
Upper Pons	11	24.96	9.64	25.80	8.68	0.84	26.11	9.70	30.57	10.63	0.40
Lower Pons	17	41.54	18.20	38.66	12.11	0.53	38.47	13.63	37.89	11.75	0.89
Cerebellar White Matter	15	46.88	18.83	41.98	12.24	0.44	46.66	16.29	40.63	9.61	0.17
Cerebellar Posterior Lobe	15	41.26	12.45	37.85	21.22	0.61	44.40	13.48	40.45	15.92	0.41

*^1^Number of patient-control pairs.*

**Significant after correction for multiple comparisons by using false-discovery rate (FDR).

*p < 0.05 but not significant after FDR correction.

Spearman’s correlation test revealed significant correlations between altered qMRI parameters and clinically observed MDS-UPDRS scores in our patients: There were significant correlations between MDS-UPDRS part I and relative proton density (Table 6) in contralateral prefrontal subcortical area (*R* = −0.458, *p* = 0.042), in bilateral splenium corporis callosi (*R* = 0.468 and 0.472, *p* = 0.038 and 0.042, respectively), and T1 in contralateral splenium corporis callosi (*R* = 0.468, *p* = 0.038), between MDS-UPDRS part II and T1 in contralateral prefrontal subcortical area (*R* = 0.509, *p* = 0.026), and relative proton density in contralateral nucleus caudatus (*R* = −0.492, *p* = 0.028), between MDS-UPDRS part IV as well as total MDS-UPSRS and T1 in contralateral prefrontal subcortical area (*R* = 0.475 and 0.555, *p* = 0.040 and 0.014, respectively) (T1 correlations not shown).

**TABLE 6:** Correlations of MDS-UPDRS to significant changes of relative proton density in respect to the most affected body side of the patients estimated by Spearman’s correlation test^1^.

Clinical scores	Ipsilateral																
		Splenium Corporis Callosi		Nucleus Caudatus		Putamen		Globus Pallidus		Thalamus		Mesencephalon					
							
	*N*	*R*	*p*	*R*	*p*	*R*	*P*	*R*	*p*	*R*	*p*	*R*	*p*				
UPDRS I	20	–0.072	0.763	–0.179	0.449	–0.165	0.487	–0.160	0.499	–0.062	0.795	–0.068	0.775				
UPDRS II	20	–0.053	0.824	–0.232	0.326	–0.273	0.244	–0.130	0.584	0.033	0.889	0.108	0.650				
UPDRS III	20	–0.109	0.649	0.059	0.805	0.319	0.170	0.391	0.088	0.279	0.234	0.121	0.612				
UPDRS IV	20	–0.084	0.725	–0.156	0.511	–0.270	0.249	–0.288	0.217	0.102	0.668	0.038	0.875				
UPDRS	20	–0.211	0.371	–0.149	0.530	0.001	0.997	0.127	0.595	0.201	0.396	0.098	0.681				

	**Contralateral**																
		**Prefrontal Subcortical Area**		**Splenium Corporis Callosi**		**Nucleus Caudatus**		**Putamen**		**Thalamus**		**Mesencephalon**		**Upper Pons**		**Lower Pons**	
								
	***N***	***R***	***p***	***R***	***p***	***R***	***P***	***R***	***p***	***R***	***p***	***R***	***p***	***R***	***p***	***R***	***p***

UPDRS I	20	–0.458	0.042*	–0.170	0.473	–0.372	0.106	–0.335	0.148	–0.234	0.321	–0.202	0.393	–0.110	0.645	–0.085	0.722
UPDRS II	20	–0.055	0.819	–0.174	0.463	–0.492	0.028*	–0.357	0.123	–0.323	0.164	0.030	0.902	–0.147	0.535	–0.036	0.882
UPDRS III	20	–0.068	0.776	0.132	0.579	0.051	0.830	–0.187	0.430	0.048	0.840	0.027	0.910	0.086	0.720	0.035	0.885
UPDRS IV	20	0.208	0.379	–0.206	0.385	–0.335	0.149	–0.308	0.187	–0.221	0.350	0.020	0.932	–0.020	0.932	–0.117	0.622
UPDRS	20	–0.149	0.530	–0.156	0.511	–0.317	0.174	–0.333	0.151	–0.164	0.489	–0.069	0.771	–0.014	0.952	0.005	0.982

*^1^12 patients with more affected right body side and 8 with more affected left body side.*

**Significant after correction for multiple comparisons by using false-discovery rate (FDR).

*p < 0.05 but not significant after FDR correction.

*Hence, only regions with significant alterations in relative proton density were used for correlational analysis.*

## Discussion

In the present study, relative proton density, T1, T2, and T2′ relaxation time were determined in early PD patients and in matched healthy controls from 28 brain regions of interest, which were selected based on their contribution to the nigro-striatal system, dopaminergic innervation or their brainstem localization. The derived values of these tissue parameters were compared between patients and controls with respect to the common asymmetric onset of PD. This allowed for the reorganization of ROIs in ipsilateral and contralateral brain hemispheres with regard to the clinical manifestation, as reported previously (Klietz et al., 2019a; Fu et al., 2020), depending on the body side with most pronounced symptoms and lateralized brain degeneration (Riederer and Sian-Hülsmann, 2012).

The most obvious brain alteration observed was the decreased relative proton density in PD patients. A decrease of proton density occurred in 19 of 28 selected ROIs (bilateral splenium corporis callosi, caudate nucleus, putamen, globus pallidus, thalamus, mesencephalon, upper and lower pons, in contralateral prefrontal cortex, and in ipsilateral primary motor area and centrum semiovale) of the patients, indicating microstructural alterations of brain tissue in these regions. The previously mentioned PD-related pathological deposits may bind water, resulting in less free water in brain tissue, which may be a reason for decreased proton density in our patients.

An additional observation was the change in brain tissue relaxation time in the PD patients. We observed that T1 relaxation time was increased in 13 of 28 ROIs (bilateral prefrontal cortex, centrum semiovale, splenium corporis callosi, putamen, in contralateral primary motor area, supplementary motor area, caudate nucleus, mesencephalon, and in ipsilateral upper pons), further, the T2 relaxation time increased in bilateral upper pons, and in ipsilateral splenium corporis callosi and cerebellar white matter, and the T2′ relaxation time increased in contralateral splenium corporis callosi and globus pallidus, and decreased in contralateral supplementary motor area and mesencephalon. While the exact underlying reason for observed parameter alterations in patients remains to be clarified, they all suggest certain PD-related changes in patients’ brain tissue.

It is interesting to look at the altered qMRI parameters in distinct brain areas in respect to their neuroanatomic function and relevance in PD. Because of the small volume of the cortical gray matter, to allow for better reproducibility, and in accordance to earlier studies of our group, we decided to measure subcortical regions adjacent to the cerebral cortex. In the subcortical areas of interest, especially in the prefrontal subcortical area, we found decreased proton density that indicates reduced free water content, and increased T1 relaxation time indicating altered spatial conformation of brain tissue molecular structures. Both were pronounced in the contralateral hemisphere. In a normal aging cortex, T1 relaxation time decreases between the third and eighth decade of life (Steen et al., 1995; Cho et al., 1997; Suzuki et al., 2006; Saito et al., 2009). In a study of Gracien et al. (2017) the cortical decrease in T1 relaxation time with age was pronounced in the dorso-frontal and temporal cortex over a timespan of 7 years. The subcortical increase of T1 relaxation time adjacent to relevant cortical areas in the PD sample in this study might be associated with the loss of meso-cortical dopaminergic innervation and degeneration of unmyelinated dopaminergic axons. Adaptive processes might include a regression of dendrites and axons, leading to a decrease in free water fraction of those areas (Freeman et al., 2008; Thiessen et al., 2013; Gracien et al., 2017). These results were contradictory to recent data of Keil et al. (2020) in a study using magnetic resonance fingerprinting, in which they reported a cortical decrease in T1 relaxation time in PD patients. However, there seems to be very limited comparability of their data with the data obtained in our study due to severe differences in the methods used and the measurement of cortical gray matter compared to subcortical white matter area, as in the present study. Interestingly, relative proton density was not significantly correlated with normal aging in cortical gray matter brain structures in a recent study by Seiler et al. (2020). Further, we did not find significant changes in T2 and T2′ relaxation time in subcortical areas, suggesting that no significant gliosis, cell loss or tissue iron depositions were present (Fatouros et al., 1991; Gelman et al., 1999, 2001; Brex et al., 2000; Lutti et al., 2014).

In the basal ganglia pathway, a significant reduction of proton density in all dopamine innervated gray matter regions was found with pronunciation of the contralateral hemisphere to the clinical PD manifestation side in PD patients. Further, a prolongation of T1 relaxation time was found in the caudate nucleus and putamen contralaterally to the clinical PD manifestation side. T2 relaxation time revealed no significant changes in the subcortical structures, however, T2′ relaxation time was significantly increased in the contralateral globus pallidus. These changes might correlate to a loss of dopaminergic innervation, aggregation of misfolded alpha-synuclein, and adaptive degeneration of neuronal dendrites with reduction of the tissue free water content, as shown for the cortical areas (Freeman et al., 2008; Thiessen et al., 2013; Gracien et al., 2017). These findings have been confirmed in animal models of experimental Parkinsonism in the striatum (Villalba and Smith, 2010, 2013).

In the brain stem area, a significant reduction of proton density in the mesencephalon and pons was found, and T1 relaxation time was prolonged in the contralateral mesencephalon of the PD patients compared to matched controls. T2 relaxation time was prolonged in the contralateral mesencephalon which might be associated with cell death of the dopaminergic neurons and gliosis in the ventral mesencephalon. Further, T2′ relaxation time was shortened in the contralateral mesencephalon, potentially linked to tissue iron accumulation. Degeneration of mesencephalic dopaminergic neurons is the main pathological hallmark of PD, and several studies investigated microstructural changes in the substantia nigra or ventral mesencephalon. A decrease in T2^∗^ relaxation time in the substantia nigra of PD patients compared to healthy controls was reported by Egger et al. (2018) however, this decrease did not reach statistical significance. Decrease in T2^∗^ relaxation time was also reported in two studies of experimental Parkinsonism in a rat model of nigro-striatal denervation (Soria et al., 2011; Fang et al., 2018). Increased iron, copper and manganese concentrations in the substantia nigra were reported in a mouse model of experimental Parkinsonism (Matusch et al., 2012). Several other studies also reported significantly increased iron concentrations in the substantia nigra of PD patients compared to healthy controls by quantitative susceptibility mapping (Langkammer et al., 2016; Wang et al., 2016; Chen et al., 2019; Cheng et al., 2020). In a study of patients with neurodegeneration with brain iron accumulation (NBIA), thalamic and mesencephalic iron accumulation was also detected (McNeill et al., 2012).

The lack of significant alterations of the T2′ sequences in the presented study of early PD patients could be due to counteracting processes in the brain stem areas. While neurodegeneration may lead to increased T2′ relaxation time, the accumulation of iron in the dopaminergic areas was shown to lead to a decrease of T2′ relaxation time (Siemonsen et al., 2008).

In the present study, no correlation between microstructural changes and PD symptoms measured by the MDS-UPDRS could be found after correction for multiple testing, despite of the distinguished profile of microstructural changes. Several other groups were also not able to find a correlation of clinical symptoms and changes in cerebral relaxometry (Nürnberger et al., 2017; Keil et al., 2020). Interestingly, a study in NBIA patients detected a significant correlation with dystonic symptoms (McNeill et al., 2012).

### Limitations

The small sample size of the carefully characterized homogeneous group of early PD patients is the major limitation of the study. While efforts were made to minimize possible bias related to natural hemisphere laterality in the human brain, it may still have some impact on the results. Larger study samples can use these preliminary data to further evaluate microstructural changes as diagnostic tests for PD. In this study, manually set ROIs were used. Atlas-based methods or statistical mapping methods would be an alternative for an objective evaluation. For general clinical application, an automated mechanism would be more suitable. Further, the effect of PD therapy on microstructural changes is unclear and should be studied in future trials.

## Conclusion

Parkinson’s disease is associated with a distinct profile of microstructural changes which may accompany relevant pathological and maladaptive processes in disease pathophysiology. The technique used in this study offers new opportunities to study distinct aspects of PD pathology *in vivo*, and might contribute to early diagnosis both as a biomarker and a parameter for treatment efficiency in future trials. Future studies should evaluate the diagnostic value of combined structural, metabolic and microstructural changes in differential diagnosis of PD and atypical Parkinsonism.

## Data Availability Statement

The raw data supporting the conclusions of this article will be made available by the authors, without undue reservation.

## Ethics Statement

The studies involving human participants were reviewed and approved by local Ethics Committee of Hannover Medical School (No. 6167-2016). The patients/participants provided their written informed consent to participate in this study.

## Author Contributions

X-QD designed the study. MK and FW were responsible for recruitment. MK and ME performed the clinical characterization of the patients. NM, MA, and PN performed the MRIs. ME and X-QD performed the statistical analysis. MK, ME, FW, and X-QD interpreted the data. MK, ME, and X-QD wrote the manuscript. NM, GH, and HL coedited the manuscript. All authors had access to the complete data generated in the study and statistical analysis, took part in the manuscript edition, and agreed to submit the manuscript for publication.

## Conflict of Interest

The authors declare that the research was conducted in the absence of any commercial or financial relationships that could be construed as a potential conflict of interest.

## Publisher’s Note

All claims expressed in this article are solely those of the authors and do not necessarily represent those of their affiliated organizations, or those of the publisher, the editors and the reviewers. Any product that may be evaluated in this article, or claim that may be made by its manufacturer, is not guaranteed or endorsed by the publisher.
